# A Preliminary Report on the Transthelial Approach to Breast Implant Exchange

**DOI:** 10.1093/asjof/ojag017

**Published:** 2026-05-04

**Authors:** J Roscoe Wasserburg, Kurt Moskovitz, Ryan Bender, Samir Hasan, Krishna Vyas, Kevin Tehrani

## Abstract

Augmentation mammoplasty is a popular aesthetic surgical procedure with over 300,000 cases performed in the United States annually, making it the second most common aesthetic surgical procedure after liposuction. Although traditional approaches to implant exchange can result in noticeable scarring, the transthelial approach offers scar concealment and direct implant access which may be advantageous for scar-averse patients. This article describes a single surgeon's preliminary experience with the transthelial approach to breast implant exchange. A retrospective chart review was performed from a single board-certified plastic surgeon to identify patients who had undergone implant exchange with a transthelial approach. Six patients were identified who underwent breast implant exchange through the transthelial approach between 2017 and 2020. All patients previously underwent saline-based implant placement through a transumbilical approach. The cohort's median age was 32 (27-50), with a median BMI of 20.4 (19.4-22.3). All patients reported satisfaction with their scars, maintenance of nipple–areolar complex sensation, and preserved vascularity. One patient (14%) had recurrence of Baker Grade IV capsular contracture which improved with nonoperative management. There were no reports of implant failure, hematoma, seroma, infection, malposition, delayed wound healing, or reoperations in this cohort. The transthelial approach is a viable option for breast implant exchange in select patients. It has the advantage of scar concealment in a naturally striated and irregular tissue, preferable for scar concealment. To obtain more conclusive and generalizable data, larger studies with longer follow-up periods are encouraged.

**Level of Evidence**: 4 (Therapeutic) 
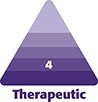

Augmentation mammoplasty, colloquially known as breast augmentation, is a popular aesthetic surgical procedure with 304,181 procedures performed in the United States in 2023, placing it second most frequent after liposuction.^1^ It is recommended that breast implants be replaced every 10 to 15 years; however, many patients will replace them earlier because of patient preference, aesthetic considerations, or complications.^2^ Breast implant removal or exchange procedures are quite common, with 41,115 patients undergoing this procedure in 2023. The primary objective of breast augmentation is to enlarge the size of the breasts and improve overall breast shape as well as to improve the position of the nipple–areolar complex (NAC).^3^

Several operative techniques for primary breast augmentation have been described, including the transaxillary, transumbilical, inframammary fold (IMF), periareolar, and transthelial approaches.^4-18^ Many surgeons will prefer to reuse previous incisions for exchange procedures; however, this is not a feasible option for patients who underwent a transumbilical approach for their primary procedure. Hence, there is a need for discussion regarding an optimal surgical approach for this patient population. Although the transthelial approach has been previously described, there exists a paucity of literature describing this technique through the lens of individual surgeon experience with outcomes data.^15,19,20^ This case series highlights the positive aesthetic outcomes and low complication rates in a cohort within the United States.^12,15,19,21^

Although there exists a consistent demand among women for breast augmentation, the perspective of what is considered an “acceptable” scar continues to change.^22^ In particular, because East Asian women have become a growing demographic seeking breast augmentation, patient preferences have shifted.^23-25^ In the United States, the Asian population has represented ∼6% of the total aesthetic plastic surgery procedures since 2015.^1^ Internationally, aesthetic surgery has increased in popularity, particularly in China, Korea, Japan, and India.^26-29^

The transthelial approach for breast augmentation has several distinct advantages including concealment of scar in naturally darker pigmented and irregular tissue as well as direct access to the implant for expeditious removal and replacement. The approach shares advantages with other implant exchange approaches, namely in ease of access to the implant. Disadvantages of this approach include potential changes to sensation of the NAC, infection, scarring, and vascular compromise. The approach may similarly be limited by patient factors as NAC diameter must be appropriate to allow for exchange.

This study examines the use of the transthelial approach to breast implant exchange at a single board-certified plastic surgeon's practice. We describe the technique, our experience in performing it, and review of existing literature on the topic.

## METHODS

A retrospective chart review was performed utilizing an electronic medical record system from a single board-certified plastic surgeon's office to review patients who had undergone implant exchange with a transthelial approach. All patients had saline implants placed in the submuscular plane at the time of their primary breast augmentation surgery through a transumbilical approach.

### Patients and Indications for Implant Exchange

From 2017 to 2020, 6 patients from a single surgeon's office underwent breast implant exchange through a transthelial approach. Four implant exchanges were secondary to capsular contracture, 1 secondary to implant deflation, and 1 secondary to undesired aesthetic outcome. For all 6 patients, both primary mammoplasty and implant exchange were performed by the same surgeon. All patients had primary breast augmentation with smooth round, saline implants in the subpectoral plane. Regarding projection, 2 patients chose implants with low profile, 2 selected a moderate profile and 2 chose high profile. Table 1 describes the demographic characteristics.

**Table 1.** Demographic Characteristics of Recruited Study Participants (*n* = 6)

Total, *n*	6	
Age, median (range)	32	(27—50)
BMI, median (range)	20.4	(19.4 - 22.3)
Smoking history, *n* (%)		
Never smoked	5	(83)
Current	1	(17)
Fitzpatrick skin type		
Fitzpatrick 2	3	(50)
Fitzpatrick 3	2	(33)
Fitzpatrick 4	1	(17)
Primary implant type, *n* (%)		
Saline	6	(100)
Primary implant texture, *n* (%)		
Smooth round	6	(100)
Primary implant profile, *n* (%)		
Low		(33)
Moderate	2	(33)
High	2	(33)
Primary implant size, median (range)	230	(210 - 360)
Primary implant technique, *n* (%)		
Transumbilical	6	(100)
Capsular contracture after primary breast augmentation, *n* (%)		
Yes	4	(67)
No	2	(33)
Primary diagnosis, *n* (%)		
Capsular contracture	4	(67)
Deflation	1	(17)
Undesired look	1	(17)

### Operative Technique

**Figure 1.**
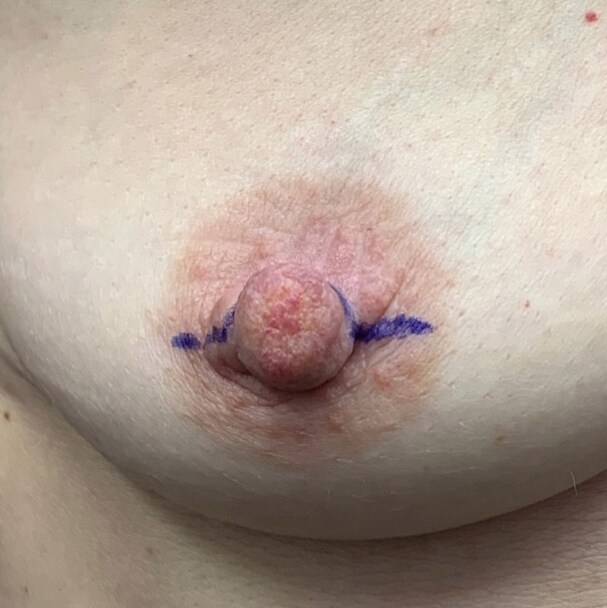
Image of a 46-year-old patient marked preoperatively with “Omega” incision pattern. Marking is from 3 o’clock to 9 o’clock position horizontally and carried along the superior border of the nipple.

The transthelial incision is marked with the patient standing, starting in a transverse direction from the 3 to 9 o’clock position of the areola or angled obliquely, and the incision is then continued along the superior or inferior edge of the nipple in the central areola (Figure 1). The medial and lateral areola extensions can also be performed in a zigzag pattern to extend the length of the incision for greater access for dissection in the setting of a small areola. As demonstrated in Video 1, the patient is prepped and draped in the usual sterile fashion in supine position. An incision is made using a 15-blade scalpel. The dissection is carried down subcutaneously and through glandular and pectoralis muscle to the level of the capsule with electrocautery, then horizontal capsulotomy is performed. The breast implant is removed from the pocket, and internal capsulotomies or capsulorrhaphies may be performed as needed in the radial and sagittal planes (Video).

Triple-antibiotic and betadine solution, followed by Phase I hypochlorous acid are used to irrigate the breast pocket. Meticulous hemostasis is obtained with electrocautery. The new breast implants are delivered into the breast pocket using a Keller funnel and no-touch technique. Closure is performed with a 2-0 Vicryl in the glandular plane and 3-0 Vicryl in the deep dermal plane. A 5-0 fast absorbing gut suture is used to approximate the halves of the NAC. Aquacel Ag dressings are applied to cover the closure over bolstered Telfa dressings.

### Assessment

Patients were asked upon office follow-up to describe their satisfaction with the scar appearance and nipple sensation. Documentation was recorded in patient charts to support subjective assessment of scar satisfaction and nipple sensation. Vascularity was considered a binary outcome of lack of or presence of preserved vascularity.

## RESULTS

Our patient cohort is reported in Table 1. Patients ranged in age from 27 to 50 years with a median age of 32 years. All had a BMI ranging between 19.4 and 22.3 with a median of 20.4 kg/m^2^. One patient endorsed active smoking, averaging <1 pack per day of cigarettes. All primary implants were smooth, round saline. Two patients had low profile, 2 patients had moderate profile, and 2 patients had high-profile implants. The average primary implant size was 230 cc (range, 210-360 cc) and 4 of the 6 patients experienced capsular contracture. The transumbilical approach was used as the primary augmentation technique in all 6 patients.

Of the 6 patients who underwent implant exchange, 2 opted for silicone implants while the remaining 4 continued with saline implants. All secondary implants had a smooth round shell, and the profile distribution was maintained (2 low, 2 moderate, and 2 high profile). Secondary implants had a mean size of 298 cc with a range from 210 to 400 cc (Table 2).

**Table 2.** Secondary Patient Characteristics and Outcomes of Transareolar Approach to Breast Implant Exchange (*n* = 6)

Total, *n*	6	
Follow-up^a^, median (range)	9.5	(1-41)
Secondary implant type, *n* (%)		
Saline	4	(67)
Silicone	2	(33)
Secondary implant texture, *n* (%)		
Smooth round	6	(100)
Secondary implant profile, *n* (%)		
Low	2	(33)
Moderate	2	(33)
High	2	(33)
Secondary implant size, mean (range)	298	(210-360)
Use of Keller funnel, *n* (%)		
No	4	(67)
Yes	2	(33)
Irrigation, *n* (%)		
Triple antibiotic + betadine + Phase 1 hypochlorous acid	4	(67)
Triple antibiotics + betadine	2	(33)
Primary outcomes, *n* (%)		
Scar satisfaction	6	(100)
Preservation of sensation	6	(100)
Maintenance of vascularity	6	(100)
Complications, *n* (%)		
Capsular contracture^b^	1	(17)
Scaring difficulty	0	(0)
Hematoma	0	(0)
Seroma	0	(0)
Malposition	0	(0)
Reoperation	0	(0)
Implant failure	0	(0)

^a^Time to follow-up is reported in months.

^b^One patient-reported symptoms consistent with Baker Grade III/IV capsular contracture.

**Figure 2.**
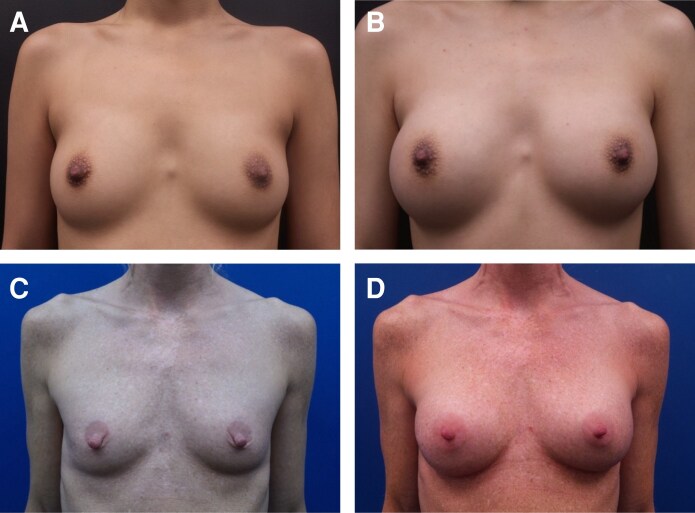
Before and after images of patients included in the study cohort. (A) Preoperative image of a 27-year-old patient. (B) Postoperative image of Patient A at 5 months. (C) Preoperative image of a 50-year-old patient. (D) Postoperative image of Patient C at 6 months.

The median time to follow-up was 9.5 months, with a range from 1 to 41 months. All patients reported satisfaction with their scars, maintenance of NAC sensation, and preserved vascularity. Postoperative photographs at 4 months (Figure 2B) and 6 months (Figure 2D) show well-healed incisions, improved projection, larger volume, and minimal scarring. There was 1 reported incidence of Grade IV capsular contracture in a patient who previously had Grade IV capsular contracture with her primary implants. This patient was treated with Aspen Ultrasound (Coral Springs, FL) which improved subjective symptoms. There were no instances of implant failure, hematoma, seroma, infection, malposition, delayed wound healing, or reoperations observed in this cohort.

## DISCUSSION

Implant-based breast augmentation is a common surgical procedure that has experienced significant innovations over the past several decades. The IMF and periareolar approaches remain the standard of care; however, these techniques can leave notable scars that are a key concern for many patients, particularly for individuals who are at higher risk of hypertrophic scarring and hyperpigmentation.^30,31^

In pursuit of scar minimization, many surgeons have explored other avenues for breast augmentation, principally the transaxillary and transumbilical (TUBA) approaches, which may require endoscopic assistance.^32-34^ These approaches necessitate an alternative incision when pocket visualization is needed, such as when capsulectomy is required.^35^ Additionally, TUBA is almost exclusively performed with saline implants.^1^ The transthelial approach offers an opportunity to minimize scar visibility on the breast and allows adequate visualization of the pocket. The procedure is a viable option for patients who seek to avoid a scar in the IMF and have adequately sized areolas to accommodate the incision. Ideal candidates for this approach include those with lightly pigmented areolas with less prominent margins, minimal ptosis, and strong preference for minimal visible scarring elsewhere on the breast.

The tendency toward developing darker, more conspicuous and hypertrophic scarring is higher in Asian populations compared with Caucasian populations.^36^ Cultural perceptions are critical for surgeons to appreciate as these play a significant role in surgical approach selection. In many Eastern cultures, any visible scarring is associated with negative perceptions of outcomes which can impact a patient's psychological well-being.^23,24^ Therefore, placing the scar in a less conspicuous area is an important consideration when performing aesthetic breast surgeries in Asian women. Because of the concern of hypertrophic scarring, the transaxillary approach is the most favored, followed by the periareolar approach. Although an inframammary incision is commonly performed in the West, it is not as widely accepted in Asia in order to avoid visible scarring on the inferior breast footprint.^24^

Nipple sensation after augmentation is self-reported as highly important for patient satisfaction, with patients reporting unsatisfactory nipple sensation postoperatively at rates approaching 15% to 30%.^37-40^ These findings highlight the importance of selecting a surgical technique that will preserve nipple sensation and natural tissue characteristics, directly impacting patient satisfaction and quality of life. Furthermore, patients value maintenance of normal tissue texture in studies examining long-term breast augmentation outcomes.^41,42^ The ideal surgical outcome would include minimal scar formation that is visually imperceptible, with maintenance of normal sensation and tissue texture, and no disruption of vascularity.

It has been reported that the periareolar approach increases the risk of capsular contracture compared with the inframammary and transaxillary approaches.^43^ In addition, there have been reports of higher satisfaction scores with the transaxillary approach in comparison to the inframammary approach.^44^ The transthelial approach, in comparison, has not been documented to compromise nipple or NAC sensation.^14,20^

Scarring with the transthelial approach has the advantage of concealment in a naturally striated and irregular tissue. Additionally, the pigmentation of the areola serves to better conceal scars in individuals who experience scar hyperpigmentation. Some argue that transthelial incisions may be less camouflaged because they are not at the natural breast cutaneous–areolar junction. Both periareolar and transthelial methods involve a scar that is visible at the central focal point of the breast and violate the lactiferous ducts and breast parenchyma during dissection. Although these scars are best concealed in swimwear compared with other approaches, the scar may be more noticeable in the upright position in the absence of clothing. In patients with demarcated, darkly pigmented areolas, a periareolar scar may be preferred to a transthelial scar because of the potential for scar hypopigmentation. Alternatively, patients with lightly pigmented areolas and indistinct margins may benefit from a transthelial scar because the periareolar scar may be more noticeable. The periareolar scar may also be preferable in some settings as it can be incorporated into a mastopexy skin design.

From a technical perspective, the transthelial approach allows for direct and immediate access to the implant, which minimizes tissue dissection and facilitates any adjustments that must be made to the pocket or implant capsule.

Although some surgeons cite concerns for capsular contracture or nipple sensation changes as reasons for avoiding transthelial breast augmentation, these fears have not been substantiated in the literature.^14,20^ Additionally, although postoperative infections have been a noted concern, this preliminary case series did not replicate these findings.^45^ There is a theoretically higher risk of capsular contracture with the transthelial approach secondary to infectious agents; however, several methods are employed to reduce the chances of contamination and subsequent capsular contracture. Strict aseptic technique, use of triple-antibiotic irrigation and Phase I hypochlorous acid, and minimal handling of the implant, all employed in this series, appear to provide acceptably low risk.^46,47^

Limitations of this study include the small sample size and heterogeneous indications for the transthelial approach. Further studies with larger sample sizes and longer follow-up times should be performed. Long-term studies assessing patient satisfaction and complication rates with the transthelial approach are currently limited. Future research should focus on these outcomes to determine enduring benefits and latent complications associated with this technique. There would be additional benefit for studies assessing patient-reported outcome measures that directly compare surgical approaches for implant exchange.

## CONCLUSIONS

In summary, although the periareolar and inframammary approaches should remain the gold standard for breast revision mammoplasty because of their established safety and efficacy, the transthelial approach offers a promising alternative for select patients. It combines the benefits of minimal visible scarring and adequate surgical access without compromising nipple sensation or vascularity.

## Supplementary Material

ojag017_Supplementary_Data
