# Supplementary figures and images for: A Preliminary Report on the Transthelial Approach to Breast Implant Exchange
Source: Aesthet Surg J Open Forum. 2026 May 4;8:ojag017. doi: 10.1093/asjof/ojag017 (PMC13270336; doi:10.1093/asjof/ojag017)

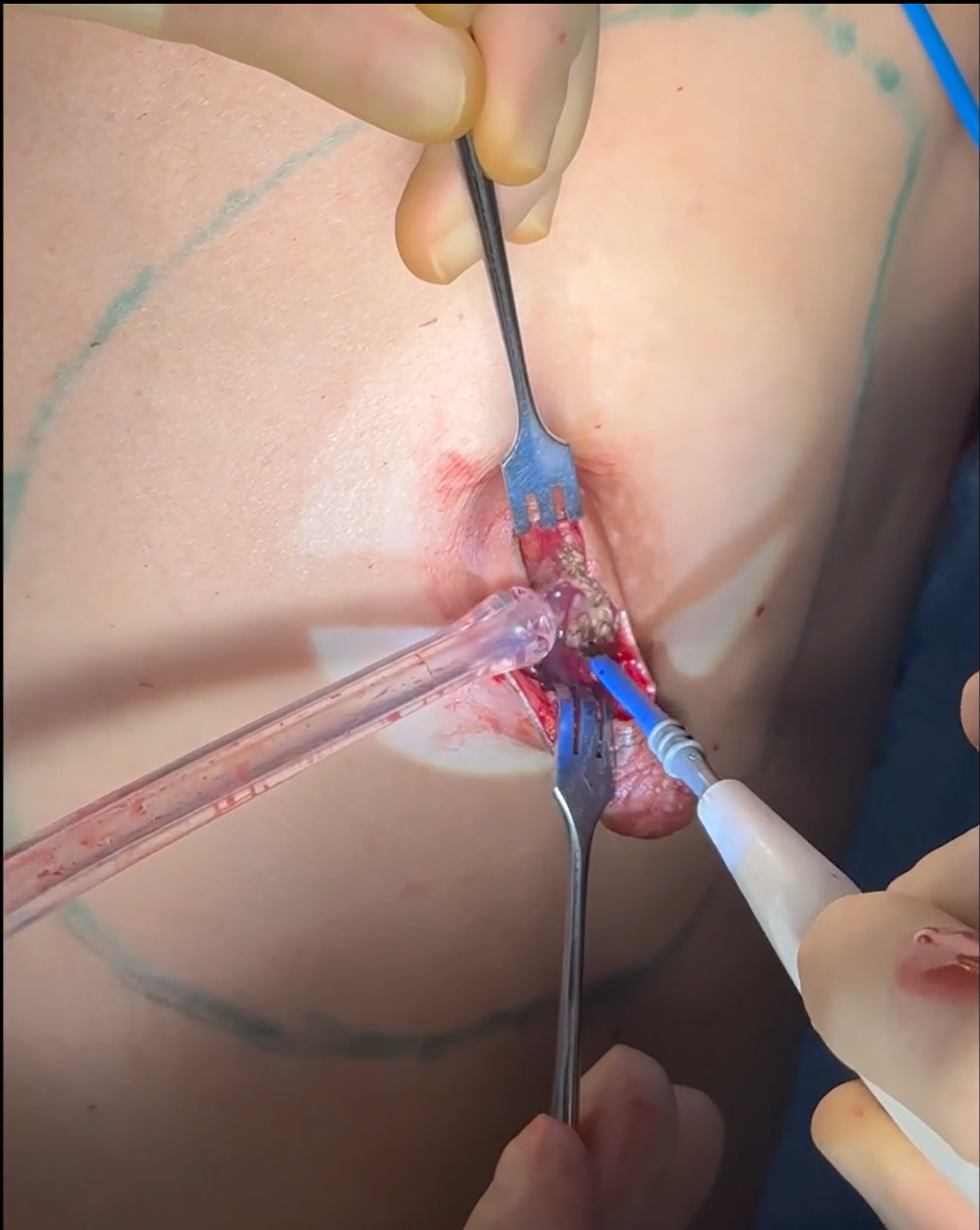

Supplement: ojag017_Supplementary_Data [file ojag017_Supplementary_Data.zip › Video still.png]
